# Resolutions by Galveston Medical Club on the Death of Dr. W. S. Rogers

**Published:** 1887-06

**Authors:** 


### SOCIETY NOTES.

### RESOLUTIONS BY GALVESTON COUNTY MEDICAL CLUB ON THE DEATH OF DR. W. S. ROGERS.

For Daniel's Texas Medical Journal.

WHEREAS it has pleased our Heavenly Father in the exercise of his inscrutable providence to remove from his sphere of usefulness, and from our companionship, the late William S. Rogers, M. D., and, Whereas, we both as a body and individually, keenly realize the loss we have sustained, we deem it our melancholy duty to give public expression to our appreciation of his high worth as a physician and a friend; therefore be it

*Resolved,* That by the death of Dr. Rogers, the Medical profession of Texas has been despoiled of an earnest supporter of every cause that seemed to tend toward the elevating of her standard, the enhancement of her usefulness, and the promoting of fellowship among those enlisted under her banner. That we remember with gratitude his distinguished services as member of the District Medical Examining Board, when his name was a terror to quacks, and an ægis to the honor of the legitimate profession. That we will cherish with pride the recollection of his admirable administration of the affairs of the Board of Health of Galveston, when he was its eminent president.

*Resolved,* That the Galveston County Medical Club has been bereft of a Fellow, whose unselfish devotion to its interests, liberal contributions to its support, and whose thoughtful counsels placed him in the front rank of its membership.

*Resolved,* That as individuals, we are painfully sensible to our loss. As a confrere he was ethical, liberal and scrupulously observant of the feelings and interests of all. As a friend he was sincere, affable, frank and generous. He never saw a fault in a friend. As a man he was brave, truthful, honest and just. Destitute of envy, he held in the highest veneration the truly meritorious, and possessed a lively appreciation of the beautiful and good, but had an innate contempt for shams. Endowed with a positive character, he had many warm friends, who loved and appreciated him; enemies also he had, but even they respected him. It can be said of him that he loved his friends and despised his enemies.

*Resolved,* That we tender the son and brother of the deceased, our heartfelt sympathy and invoke for them that consolation which can only be accorded by Him, who directeth the sparrow's fall.

*Resolved,* That these resolutions be spread upon the minutes of our club; that a copy of them be sent to the son and brother of the deceased respectively, and that copies be furnished the Galveston News and the Medical periodicals of the State for publication.

J. F. Y. PAINE, M. D.<br>

E. GOLDMANN, M. D. } Committee.<br>

H. P. COOKE, M. D.

GALVESTON, May 30, '87.

